# The Synergistic Effect of Ozonated Oil and Desensitising Toothpaste on Dentinal Tubule Occlusion: An In Vitro Study

**DOI:** 10.7759/cureus.56131

**Published:** 2024-03-13

**Authors:** Mayurakshi Saha, Sangamesh NC, S Bhuvaneshwari, Silpiranjan Mishra, Atul Anand Bajoria, Vijaylaxmi P Male

**Affiliations:** 1 Department of Oral Medicine and Radiology, Kalinga Institute of Dental Sciences, Bhubaneswar, IND; 2 Department of Oral Medicine and Radiology, Maharashtra Institute of Dental Sciences and Research, Latur, IND

**Keywords:** ozonated oil, desensitising toothpaste, scanning electron microscope, dentin hypersensitivity, dentinal tubule occlusion

## Abstract

Introduction

Dentin hypersensitivity (DH) is the most common problem encountered by clinicians. It can be managed either by blocking neural activities or by occluding tubules. Ozonated oil facilitates the simple passage of desensitizing agents into dentinal tubules.

Aim

This study aimed to evaluate the effect of ozonated oil on dentinal tubule occlusion before and after the application of desensitizing toothpaste.

Materials and methods

The study was carried out in Kalinga Institute of Dental Sciences, KIIT (Deemed to be University), Bhubaneswar, India. The sample size of the study was 80. The first group contained specimens for control. The second group comprised specimens treated with ozonated oil. The third group comprised specimens being treated with a desensitizing agent. The fourth group contained specimens treated with both the desensitizing agent and ozonated oil. The specimens then received an acid challenge. The specimens were observed under a scanning electron microscope (SEM) before any therapy, after the application of the therapeutic agents and after the 37.5% ortho-phosphoric acid challenge.

Results

Inferential statistics to compare between the groups was calculated using one-way analysis of variance (ANOVA) statistics. Post-hoc Tukey's honestly significant difference (HSD) was performed to compare the groups. The mean scores of the partial tubular occlusion of Group 1, Group 2, Group 3 and Group 4 before the acid challenge were 0.035, 0.691, 0.564 and 0.368, respectively. The maximum score was obtained in the case of Group 2, which was statistically significant. The mean scores for partial tubule occlusion after the acid challenge for Group 1, Group 2, Group 3 and Group 4 were 0.055, 0.531, 0.733 and 0.142, respectively. There was evidence of maximum partial tubule occlusion after the acid treatment in the case of Group 3. The mean scores of Group 1, Group 2, Group 3 and Group 4 before the acid challenge were 0.019, 0.309, 0.442 and 0.609, respectively. The maximum score was obtained in the case of Group 4, implying a greater number of total tubular occlusions before the acid challenge. The mean scores of the total tubular occlusions after the acid challenge for Group 1, Group 2, Group 3 and Group 4 were 0.047, 0.465, 0.272 and 0.890, respectively. There was evidence of maximum tubule occlusion in the case of Group 4, which was statistically significant.

Conclusion

Overall, the application of a desensitizing toothpaste with ozonated oil holds promise as a potentially more effective treatment approach for DH. Further research and clinical studies may be needed to fully validate its efficacy and safety in dental practice.

## Introduction

Dentin hypersensitivity (DH) is ubiquitous. Research studies investigating it have been abysmal. Unfortunately, there does not seem to be an effective or long-lasting cure for this excruciating clinical condition [1]. According to the hydrodynamic theory, an external stimulus results in fluid movement within the dentinal tubules, which deforms the odontoblastic processes and the nearby nerve fibres, causing DH [2].

The mechanism of how stimuli applied to the exterior dentin surface excite nerve fibres is yet unclear. Previous literature on prevalence studies has been inconsistent, ranging from 1.3% to 92.1% [3]. Attrition, abrasion or erosion cause the loss of tooth structure or enamel, whereas gingival recession, periodontal treatment, or poor oral hygiene can cause denudation of the root surface. DH is generally more common in females than in males and in people between the ages of 20 and 40 [4].

As of practice, the most commonly available therapeutic options include blocking the flow of fluid through the dentinal tubules or desensitising the nerve to reduce its sensitivity to stimulation [5]. Desensitisation can be accomplished using a wide range of therapies, such as solutions, gels and pastes containing fluorides in a variety of forms and concentrations. Gluma desensitiser, made by Heraeus Kulzer GmbH in Germany, contains an aqueous solution of 35% hydroxyethyl methacrylate and 5% glutaraldehyde as a desensitising agent [6]. The company Den Shield of Alachua, Florida, USA, has unveiled an item named Nova Min, made of calcium sodium phosphosilicate [7]. The process of iontophoresis acts by affecting ionic movements by electric flows, hence upgrading particle take-up by the dentinal tubules and accomplishing desensitisation. By restricting dentinal tubules inherently, glutaraldehyde prevents the hydrodynamic mechanism that causes DH [8].

The literature is scarce in providing the efficacy of ozonated oil in treating dentinal hypersensitivity. Therefore, the present study was performed to evaluate the effectiveness of ozonated oil with ozone gas. This study also evaluated the dentinal tubule occlusion before and after application with and desensitising toothpaste on dentinal tubule occlusion.

## Materials and methods

This in vitro study was executed at Kalinga Institute of Dental Sciences, KIIT (Deemed to be University), a tertiary care dental hospital in Bhubaneswar City, India. The ethical committee clearance was obtained from the Institutional Ethics Committee (Ref. No.: KIIT/KIMS/IEC/846/2022) of Kalinga Institute of Medical Sciences, Bhubaneswar, India.

Calculation of the sample size

The analysis was based on a two-sample t-test. Overall, the analysis suggested that with a total sample size of 24 (six observations per group), a significance level of 0.05 and an effect size of 2.4017507, with a high power of 0.9620982, was applied to detect a difference between the means of the two independent groups.

We considered 20 in each group based on the availability of the samples. Hence, a total of 80 samples were taken and were equally divided into four groups. The first group contained specimens for control. The second group comprised specimens treated with ozonated oil. The third group comprised specimens being treated with desensitising toothpaste (Sensodyne Deep Clean owned by Haleon). The fourth group contained specimens treated with both desensitising toothpaste and ozonated oil.

The inclusion criteria included permanent teeth indicated for therapeutic purposes, such as orthodontic treatment and extraction of impacted teeth. The exclusion criteria included carious teeth, teeth that had undergone periodontal treatment during the six months before extraction, endodontically treated teeth and fractured teeth.

Extracted teeth were cleaned to get rid of any blood or debris and then preserved in a 10% formalin solution for later use. A diamond disc bur was used to create cross-sectional dentin discs. Oval-shaped dentin discs were prepared with a maximum dimension of 1 cm having 1 mm of thickness. To generate a homogeneous surface, dentin discs were polished using sandpaper. To eliminate the polishing abrasive, the polished specimens were then sonicated using an ultrasonic cleaner. After that, ozonated oil and ozone gas were added to the dentin specimens of the second group. Desensitising toothpaste was applied to the dentin specimens in the third group. The dentin specimens in the fourth group were prepared by applying toothpaste and ozonated oil. Following preparation, the samples were examined under 2000x magnification using a scanning electron microscope (SEM).

The samples were then completely cleaned with distilled water, placed in a new tube with a 37.5% ortho-phosphoric acid solution and further sonicated for half a minute. The specimens were etched with acid, cleansed with distilled water and then placed in phosphate-buffered saline (PBS). The dentin discs went through SEM analysis. At 2000x magnification, the images of the SEM were recorded.

**Figure 1:**
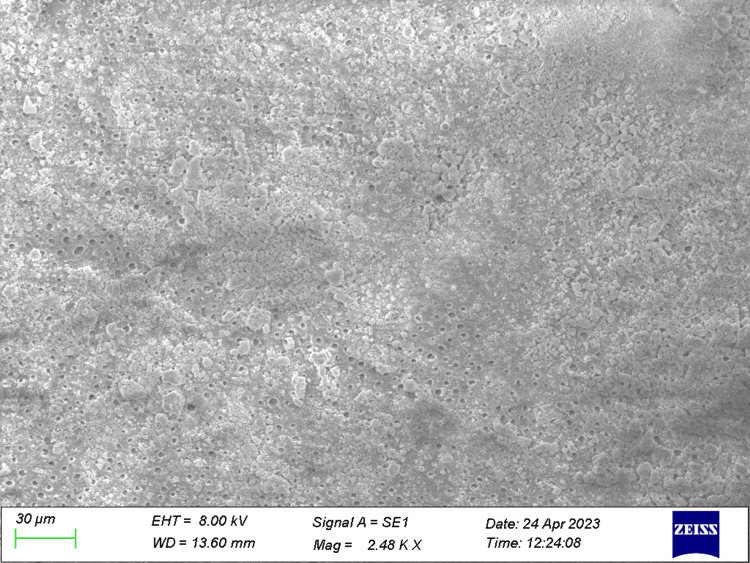
Specimen from Group 1 (control) The figure demonstrates open dentinal tubules.

**Figure 2:**
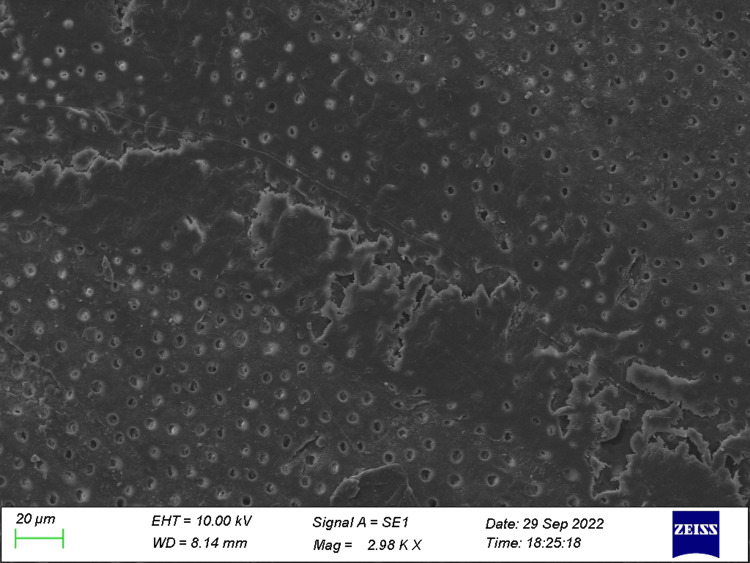
Specimen from Group 2 (before the acid challenge) The dentin specimen treated with ozonated oil prior to the acid challenge shows variation in the occlusion of dentinal tubules.

**Figure 3:**
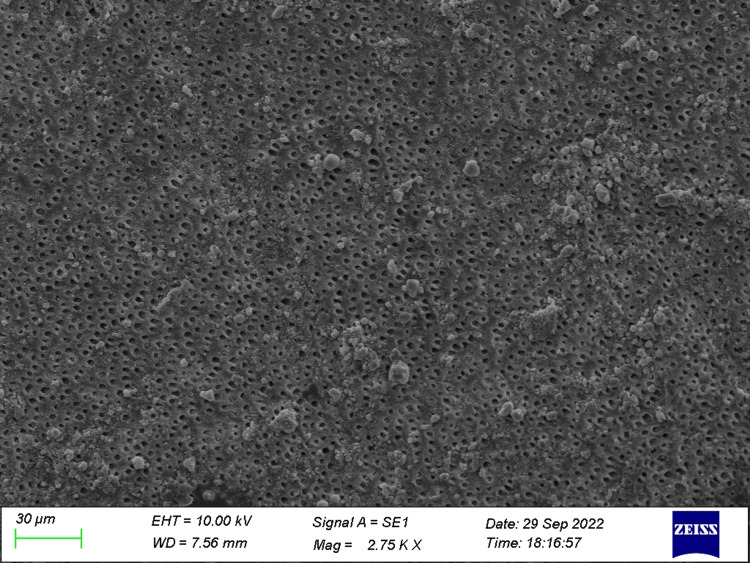
Specimen from Group 3 (before the acid challenge) The dentin specimen treated with toothpaste prior to the acid challenge shows a higher number of partial occlusion of tubules.

**Figure 4:**
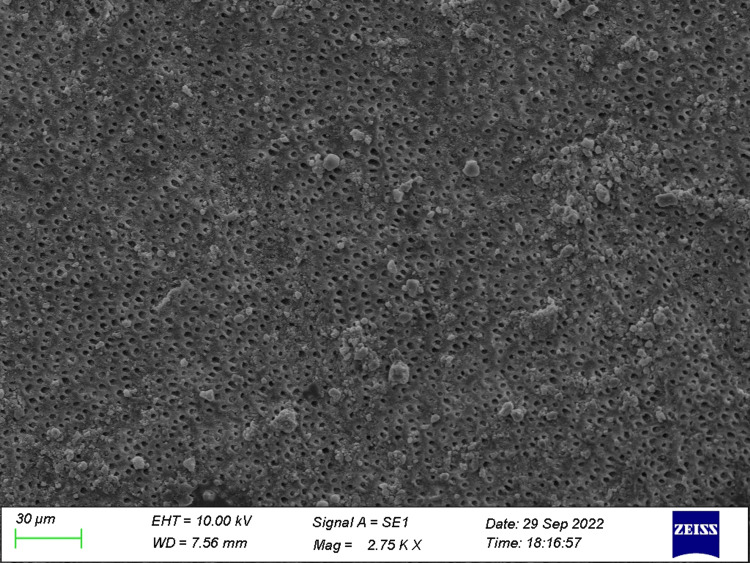
Specimen from Group 4 (before the acid challenge) The dentin specimen treated with both ozonated oil and toothpaste showed a greater number of occluded tubules.

**Figure 5:**
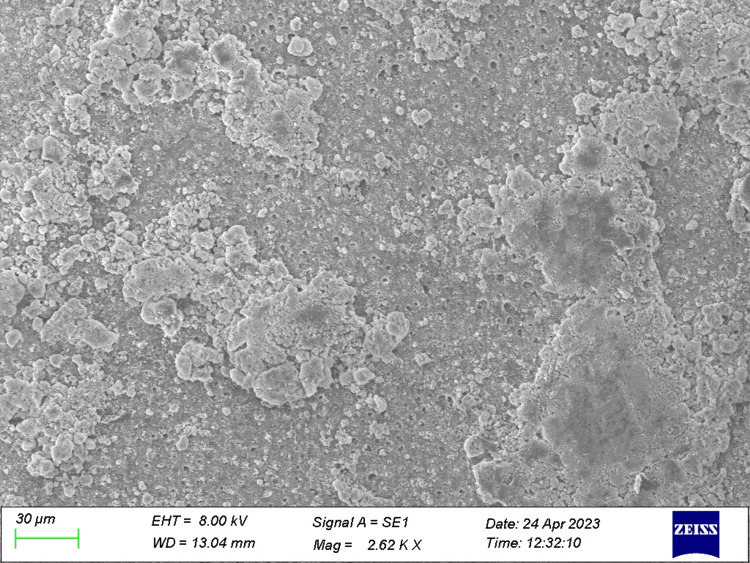
Specimen from Group 2 (after the acid challenge) The specimen treated with ozonated oil post acid challenge.

**Figure 6:**
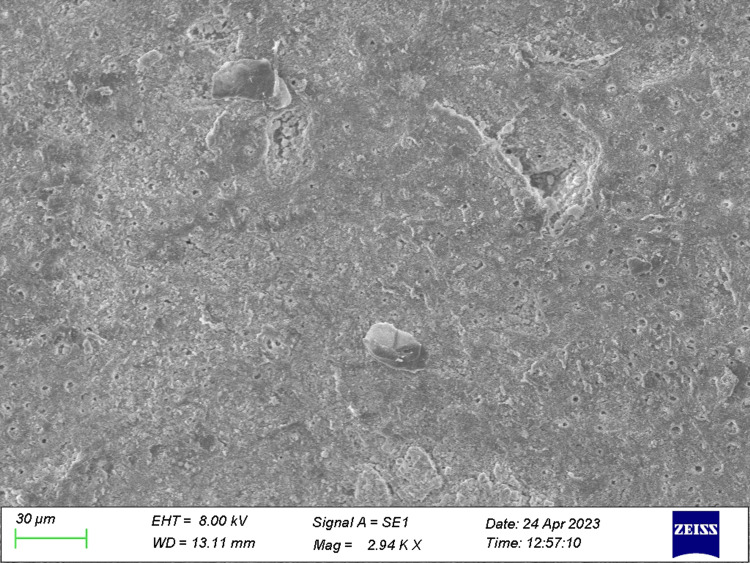
Specimen from Group 3 (after the acid challenge) The specimen treated with toothpaste post acid challenge.

**Figure 7:**
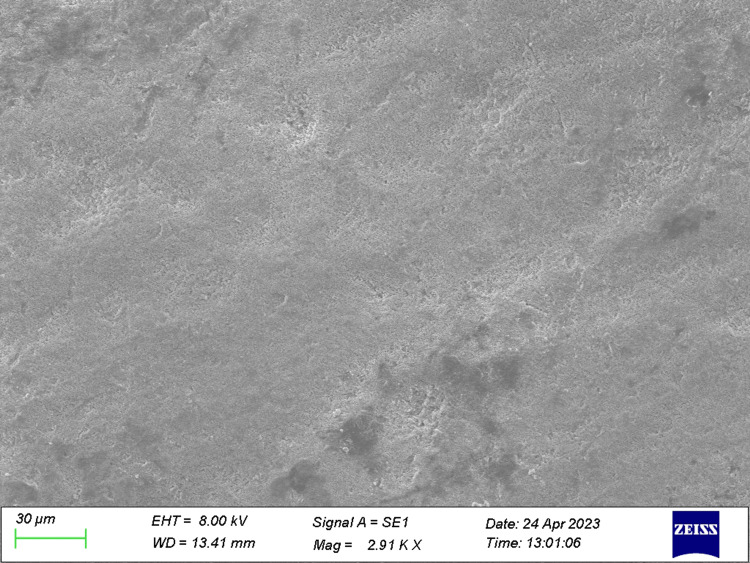
Specimen from Group 4 (after the acid challenge) The specimen treated with both ozonated oil and toothpaste post acid challenge showed a total occlusion of dentinal tubules.

The total number of tubules and the number of open and occluded tubules were individually quantified for each picture of all the specimens obtained (Figures 1-7).

To calculate the percentage of occluded tubules, the total number of occluded tubules in each picture was divided by the total number of tubules and then multiplied by 100.

MS Excel 2016 (Microsoft Corporation, USA) was utilized to enter data and analyzed using IBM SPSS Statistics for Windows, version 25 (released 2017; IBM Corp., Armonk, New York, United States) using descriptive and inferential statistics. For the continuous variables, the mean and standard deviation (SD) were calculated. Inferential statistics to compare amongst the groups was calculated using one-way ANOVA statistics, and post-hoc Turkey's honestly significant difference (HSD) was performed for comparing between the groups. P-value was kept <0.05. Any value below the aforementioned value was considered statistically significant.

## Results

The present study was performed to differentiate the efficacy of therapeutic agents on dentinal tubular occlusion. After the preparation of dentin specimens, tubular occlusion was evaluated through SEM analysis. Following manual calculation of partially occluded tubules and totally occluded tubules, the data were obtained and statistical analysis was performed.

**Figure 8:**
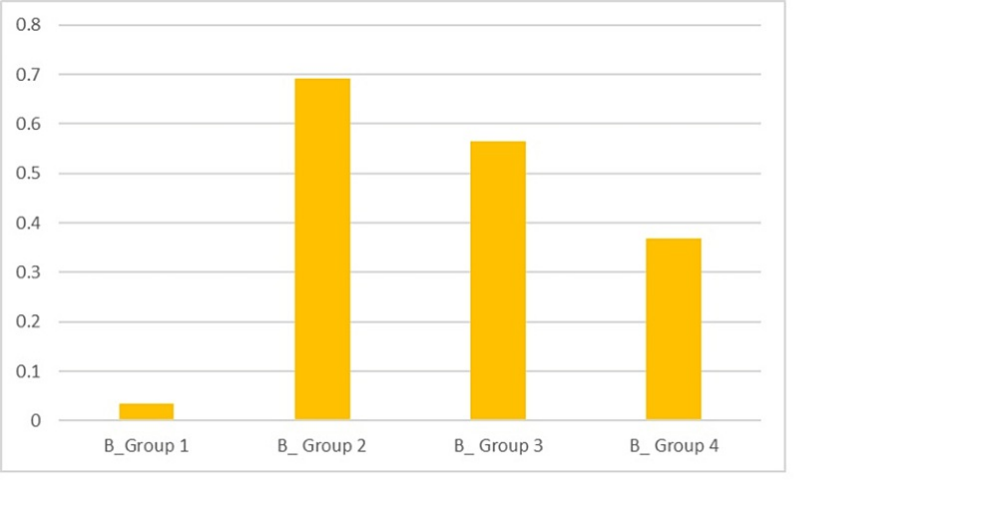
Graphical representation of the score before the acid challenge for the groups for PO (partial occlusion)

The tubules of different groups that were partially occluded were compared. The mean score calculated in the case of Group 1, which served as the control, was 0.035 before the acid challenge. The mean scores of Groups 2, 3 and 4 before the acid challenge were 0.691, 0.564 and 0.368, respectively. The maximum score was obtained in the case of Group 2, implying a greater number of partial tubular occlusion before the acid treatment, which was statistically significant (P < 0.0001) (Table 1, Figure 8).

**Table 1:** Descriptive statistics for the before and after the acid treatment comparison for each group PO (partially occluded) *Statistically significant

PO	Mean	Std. deviation	Mean difference	SD	P-value
B_Group 1	0.035	0.0041	-0.019	0.0037	<0.0001*
A_ Group 1	0.055	0.0038			
B_ Group 2	0.691	0.1446	0.160	0.1463	<0.0001*
A_ Group 2	0.531	0.0150			
B_ Group 3	0.564	0.0138	-0.168	0.0254	<0.0001*
A_ Group 3	0.733	0.0186			
B_ Group 4	0.368	0.0414	0.225	0.0419	<0.0001*
A_ Group 4	0.142	0.0055			

**Figure 9:**
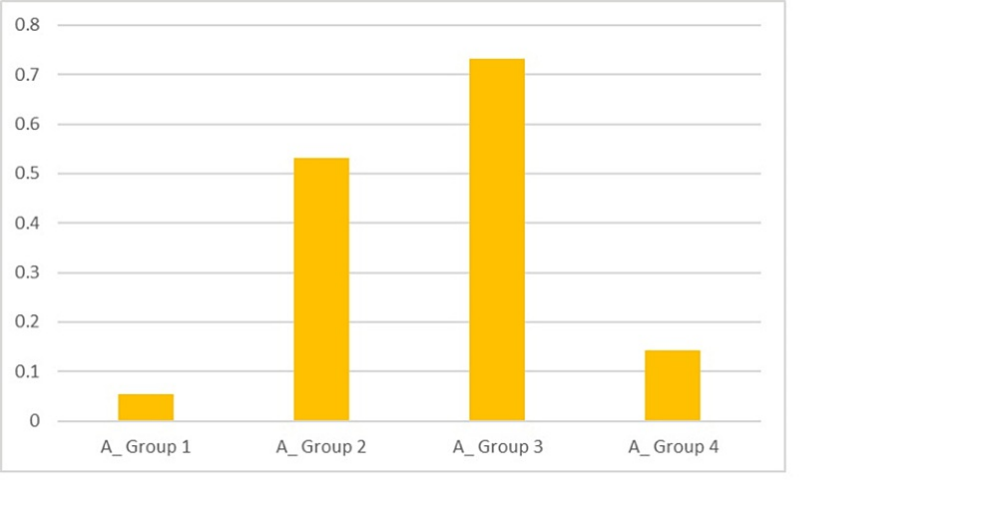
Graphical representation of the scores after the acid challenge for the groups for PO (partial occlusion)

The mean scores obtained, as depicted in Table 1, for Groups 1, 2, 3 and 4 were 0.055, 0.531, 0.733 and 0.142, respectively, after the acid challenge. There was evidence of maximum partial tubule occlusion after the acid treatment in the case of Group 3, which was quantified as statistically significant (P < 0.0001) (Figure 9).

**Figure 10:**
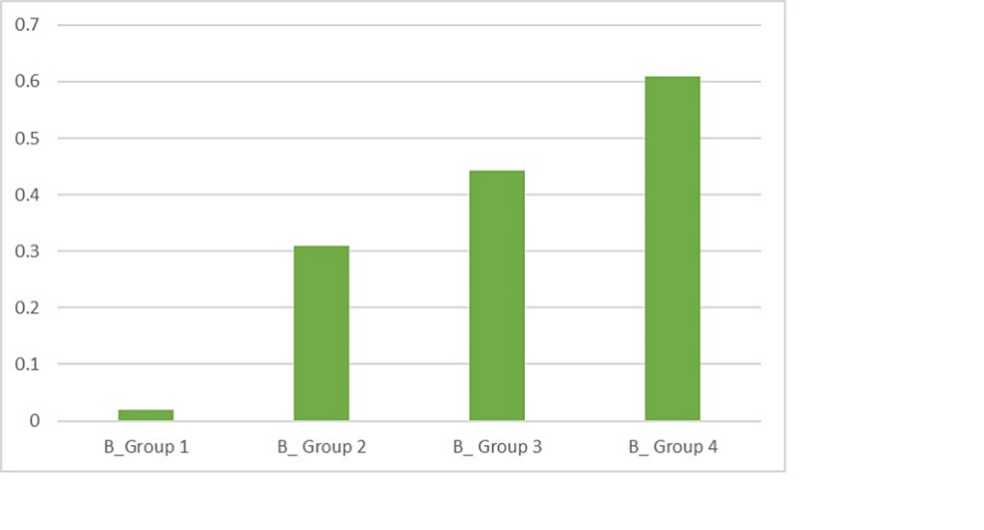
Graphical representation of the before acid challenge for the groups for TO (total occlusion)

The totally occluded tubules of different groups were calculated. Prior to the acid challenge, the mean scores for Groups 1, 2, 3 and 4 were, respectively, 0.019, 0.309, 0.442 and 0.609. The maximum score was obtained in the case of Group 4, implying a greater number of tubular occlusion before the acid challenge. It was considered statistically significant (P < 0.0001) (Table 2, Figure 10).

**Table 2:** Descriptive statistics for the before and after acid challenge comparison for each group (total occlusion) *Statistically significant

TO	Mean	Std. deviation	Mean difference	SD	P-value
B_Group 1	0.019	0.0065	-0.0275	0.007	<0.0001*
A_ Group 1	0.047	0.0005			
B_ Group 2	0.309	0.1502	-0.156	0.150	<0.0001*
A_ Group 2	0.465	0.0174			
B_ Group 3	0.442	0.0439	0.170	0.045	<0.0001*
A_ Group 3	0.272	0.0087			
B_ Group 4	0.609	0.0824	-0.281	0.089	<0.0001*
A_ Group 4	0.890	0.0141			

**Figure 11:**
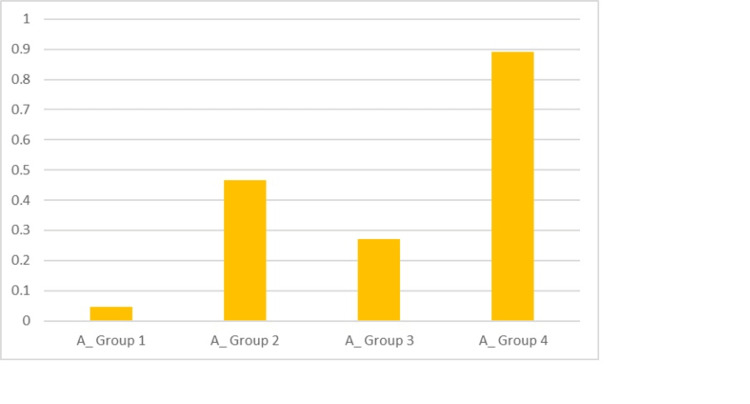
Graphical representation of the scores after the acid challenge for the groups for TO (total occlusion)

Following the acid challenge, the mean scores as depicted in Table 2 for Groups 1, 2, 3 and 4 were, respectively, 0.047, 0.465, 0.272 and 0.890. There was evidence of maximum tubule occlusion in the case of Group 4, which was determined as statistically significant (P < 0.0001) (Figure 11).

Multiple-group comparison of total tubule occlusion

Multiple-group comparison was made, and there was a statistically significant difference between the groups for individual-group comparison for the total occlusion (TO) group.

The mean difference values on comparing Group 1 with Groups 2, 3 and 4 obtained were -0.4187, -0.2255 and -0.8433. Similarly, comparing Group 2 with Groups 1, 3 and 4, the values obtained were as follows: 0.4187, 0.1932 and -0.4245. On comparing Group 3 with Groups 1, 2 and 4, the scores were -0.1932, -0.6177 and 0.8433.

The comparison of Group 4 with Groups 1, 2 and 3 resulted in the following scores: 0.8433, 0.4245 and 0.6177 (Table 3). The standard error was 0.0038. Hence, the maximum score was obtained by Group 4.

**Table 3:** Descriptive statistics for the comparison among each group using post-hoc Turkey's honestly significant difference (HSD). *The mean difference is significant at the 0.05 level (*statistically significant).

	(I) Subgroup	(J) Subgroup (dependent variable)	Mean difference (I-J)	Std. error	P-value	95% confidence interval	
						Lower bound	Upper bound
TO (multiple comparisons)	1	2	-0.4187	0.0038	<0.0001*	-0.4288	-0.4088
		3	-0.2255	0.0038	<0.0001*	-0.2356	-0.2156
		4	-0.8433	0.0038	<0.0001*	-0.8533	-0.8333
	2	1	0.4187	0.0038	<0.0001*	0.4088	0.4288
		3	0.1932	0.0038	<0.0001*	0.1832	0.2032
		4	-0.4245	0.0038	<0.0001*	-0.4346	-0.4145
	3	1	0.2255	0.0038	<0.0001*	0.2156	0.2356
		2	-0.1932	0.0038	<0.0001*	-0.2032	-0.1832
		4	-0.6177	0.0038	<0.0001*	-0.6278	-0.6077
	4	1	0.8433	0.0038	<0.0001*	0.8333	0.8533
		2	0.4245	0.0038	<0.0001*	0.4145	0.4346
		3	0.6177	0.0038	<0.0001*	0.6077	0.6278

## Discussion

One of the most vexing diseases in clinical practice is DH. The treatments available are highly effective yet scarce. This can cause both physical and psychological problems for the patient [9]. The use of toothpaste containing potassium nitrate and fluoride has a positive effect in counteracting DH [10]. Several studies have also shown that remineralising toothpastes containing sodium fluoride and calcium phosphates can significantly decimate DH [11]. Potassium salts (potassium nitrate) coupled with a laser can be placed between nerve impulse transmission interruptions. The use of an Er:YAG laser with a frequency of 3 Hz and an energy of 100 mJ was seen to be effective in treating DH [12]. In our study, we used potassium nitrate-containing toothpaste (Sensodyne Deep Clean owned by Haleon).

The exact role of ozone in DH is still controversial. Abdelaziz et al. [13] stated about the removal of the smear layer by ozonation in exposed teeth which led to the opening of dentin tubules, thereby increasing their diameter and facilitating mineral absorption. They conducted a study to assess the impact of ozone. The collaborative use of ozone and fluoride culminated in a significantly higher percentage of tubular occlusion compared to fluoride desensitiser solely. Our results also concluded the efficacy of the combined use of ozonated oil and ozone gas along with potassium nitrate-containing toothpaste in treating DH.

Addy et al. [14] conducted a study that showed Crest (NaF) to be more effective in treating dentin sensitivity. Arrais et al. [15] in their study found Oxa-Gel to be a better desensitising agent. Wang et al. [16] used Sensodyne, which showed significant resistance to acid attacks. Rajesh et al. [17] found dentifrice containing 5% Novamin® to be significantly more effective in reducing DH. Torres et al. [18] showed that Admira Protect and Bifluorid 12 cemented a longer-lasting desensitising effect compared to Colgate Pro-Relief in-office. The present study demonstrated the maximum number of partial tubular occlusions in dentin specimens treated with Sensodyne toothpaste containing potassium nitrate. Similar results were corroborated in studies conducted by Arrais et al. and Wang et al. This theory is based on the phenomenon of increased potassium ion concentration ([K+ ]) in extracellular fluids into tubules causing sustained nerve depolarisation. A significant amount of reduction in permeability was noted even after a citric acid challenge just like the study conducted by Wang et al. The present study's results do not correlate with the results of Addy et al., Rajesh et al. and Torres et al. The variation in results may be due to different compositions of toothpastes and ethnicity.

Veena et al. [19] found effective tubular occlusion when ozonated oil was used in conjunction with a desensitising agent containing arginine. In the present study, we evaluated total and partial occlusions of dentinal tubules independently. The majority of tubules in the control group were found to be unoccluded, with a smear layer occluded in some of them. By contrast, the majority of tubules in the specimens treated with desensitising toothpaste, ozone oil and both therapeutic agents were found to be partially or completely occluded. These treatments operate on the phenomenon of tubule occlusion by the utilisation of therapeutic agents' infiltration. In our study, after initial application, ozone oil and ozone gas produced a greater number of partially occluded tubules before the acid challenge, while in the case of specimens treated with desensitising toothpaste, a greater number of partially occluded tubules after the acid challenge was depicted; in both cases, the results were statistically significant. The maximum number of the TO of dentinal tubules was manifested in dentin discs treated with both ozonated oil and desensitising toothpaste prior to and post acid challenge. The tubules were occluded with crystal-like precipitates. Ozone's potent oxidising ability aids in the management of dentinal hypersensitivity. When exposed dentin is treated with ozone, smear layers are removed, dentinal tubules are opened up, their width is expanded and mineral entry is made easier.

However, the limited size of the study might act as a constraint in producing optimum results. Our study was confined to only potassium nitrate dentrifices. The use of fluoride-containing toothpaste would have produced better results. The duration of the study was one year, and it was devoid of long-term follow-up.

## Conclusions

The phenomenon of compact deposition of desensitising agent particles when ozonated oil is applied adjunctively suggests a potential synergistic effect. Based on the investigations, it is reasonable to infer that combining these two agents may offer significant therapeutic benefits in managing DH.

However, further research, including clinical trials, would be necessary to validate this hypothesis and determine the extent of the therapeutic benefits provided by the combined application of ozonated oil and a desensitising toothpaste in treating DH.
